# A systems biology approach to investigate the effect of pH-induced gene regulation on solvent production by Clostridium acetobutylicum in continuous culture

**DOI:** 10.1186/1752-0509-5-10

**Published:** 2011-01-19

**Authors:** Sylvia Haus, Sara Jabbari, Thomas Millat, Holger Janssen, Ralf-Jörg Fischer, Hubert Bahl, John R King, Olaf Wolkenhauer

**Affiliations:** 1 University of Rostock, Institute of Computer Science, Department of Systems Biology & Bioinformatics, 18051 Rostock, Germany; 2 University of Nottingham, School of Mathematical Sciences, University Park, Nottingham, NG7 2RD, UK; 3 University of Rostock, Institute of Biological Sciences, Division of Microbiology, 18051 Rostock, Germany; 4 Stellenbosch Institute for Advanced Study, Stellenbosch 7600, South Africa

## Abstract

**Background:**

*Clostridium acetobutylicum *is an anaerobic bacterium which is known for its solvent-producing capabilities, namely regarding the bulk chemicals acetone and butanol, the latter being a highly efficient biofuel. For butanol production by *C. acetobutylicum *to be optimized and exploited on an industrial scale, the effect of pH-induced gene regulation on solvent production by *C. acetobutylicum *in continuous culture must be understood as fully as possible.

**Results:**

We present an ordinary differential equation model combining the metabolic network governing solvent production with regulation at the genetic level of the enzymes required for this process. Parameterizing the model with experimental data from continuous culture, we demonstrate the influence of pH upon fermentation products: at high pH (pH 5.7) acids are the dominant product while at low pH (pH 4.5) this switches to solvents. Through steady-state analyses of the model we focus our investigations on how alteration in gene expression of *C. acetobutylicum *could be exploited to increase butanol yield in a continuous culture fermentation.

**Conclusions:**

Incorporating gene regulation into the model of solvent production by *C. acetobutylicum *enables an accurate representation of the pH-induced switch to solvent production to be obtained and theoretical investigations of possible synthetic-biology approaches to be pursued. Steady-state analyses suggest that, to increase butanol yield, alterations in the expression of single solvent-associated genes are insufficient; a more complex approach targeting two or more genes is required.

## Background

A renewed interest in the development of biofuels is emerging as a result of a variety of factors including dwindling crude oil reserves, concerns over the environmental impact of fossil fuels and threats to national security potentially limiting access to resources [1]. In recent years, biofuels have been predominantly sourced from crops, resulting in competition for limited food resources and land [2-5]; bacterial fermentation has been considered a possible answer to this problem [6]. One of the best-studied bacteria for biofuel production is *Clostridium acetobutylicum*. Clostridial bacteria are strictly anaerobic, Gram-positive and form highly-resistant spores. Many of the clostridial species, such as *Clostridium difficile *and *Clostridium botulinum*, are highly pathogenic and cause devastating diseases. Some, however, like *C. acetobutylicum *which was first isolated from corn in 1912 by Chaim Weizmann [7], are harmless to humans, animals and plants and make a wide range of useful chemicals [8].

The metabolism of *C. acetobutylicum *is characterized by the so called acetone-butanol fermentation (AB fermentation) which is also referred to as acetone-butanol-ethanol (ABE) fermentation. Since butanol is a more efficient biofuel than many other solvents such as ethanol, much research is currently focused on this bacterium [1,9]. The metabolic pathway of AB fermentation comprises two characteristic phases: acidogenesis and solventogenesis. During the transition phase the generation of the solvents acetone and butanol is induced and these become the dominant fermentation products. This switch is called the metabolic (or solventogenic) shift. Though the metabolic pathways leading to solvent and acid production are clearly defined [10], the mechanisms governing the shift are not well understood. It has been shown, however, that under continuous culture conditions in a chemostat, the external pH is a crucial prerequisite for the induction of this metabolic change [11,12].

**Figure 1:**
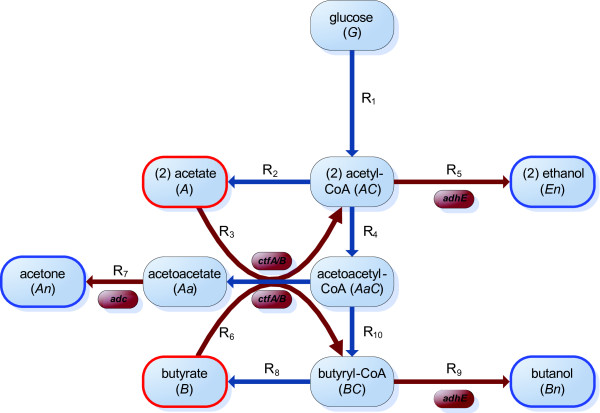
**A schematic view of the joint metabolic and gene regulation network model of AB fermentation in *C. acetobutylicum***. Acids are outlined in red and solvents in blue. The AB fermentation is characterized by a bi-phase metabolism. Following glycolysis the cells produce either the acids acetate (*A*) and butyrate (*B*) at a high pH, or the solvents acetone (*An*) and butanol (*Bn*) at a low pH while ethanol (*En*) is made in both phases but at a relatively low level. A characteristic reaction in this metabolism is the conversion of acetate (or butyrate) and acetoacetyl-CoA into acetyl-CoA (or butyryl-CoA) and acetoacetate, this being the first step in the formation of solvents from acids. We reduced the metabolic network published in [10] to ten reactions *R_i _*found in Table 1. For reactions *R*_3_, *R*_5_, *R*_6_, *R*_7_, and *R*_10 _we include gene regulation for the enzymes which are involved in the production of the solvents.

Glucose transport into the cell is mediated by a phosphoenolpyruvate-dependent phosphotransferase system. Intracellular processing via glycolysis follows the formation of pyruvate as a central metabolite [10]. A reduced metabolic network of AB fermentation is shown in Figure 1. Acetyl-CoA (which will be denoted *AC *in our mathematical model) produced from pyruvate is a branch-point intermediate located at the node dividing the pathways, one leading to the formation of the acids acetate (*A*) and butyrate (*B*), and the other to the solvents acetone (*An*), butanol (*Bn*) and ethanol (*En*). In the acid producing branches, acetyl-CoA and butyryl-CoA (*BC*) are first converted to acetyl phosphate and butyryl phosphate, respectively; these acylphosphates are then converted to acetate and butyrate (see [10] for a review). The acids are excreted from the cell and can be re-absorbed subsequently. Reinternalized butyrate and acetate molecules are converted to butyryl-CoA and acetyl-CoA respectively in Ping-Pong-Bi-Bi reactions (for more information about this type of enzymatic reaction, see for example [13]). These reactions require the two substrates (acetoacetyl-CoA, *AaC*, and butyrate or acetate) and result in two products (acetoacetate, *Aa*, and butyryl-CoA or acetyl-CoA respectively). Both reactions are mediated by the CoA-transferase which consists of the two subunits CtfA/B. Acetoacetate is converted into acetone by the enzyme acetoacetate decarboxylase, or Adc (*Ad*) [14,15]. During the solvent production phase, acetyl-CoA and butyryl-CoA are converted to acetylaldehyde and butyraldehyde, respectively, as intermediates for ethanol and butanol. Two sets of dehydrogenase activities are required to accomplish these reductions. *C. acetobutylicum *is able to ferment ethanol independently from acetone and butanol in acidogenesis under continuous culture conditions, i.e. although it is a solvent, it is produced at roughly the same quantities during acidogenesis and solventogenesis.

Experiments showed that in continuous culture *C. acetobutylicum *shifts its metabolism in response to the external pH: cells produce predominantly acetate and butyrate above a pH of 5.0 and acetone and butanol when the pH is lower than 5.0 [11,12,16,17]. It is postulated that the switch to solventogenesis represents one aspect of a survival strategy in response to external pH [1,10]. Concerning this aspect it is noteworthy that in contrast to many other organisms, clostridial bacteria seem to be unable to maintain the internal pH value at a more or less constant level. Instead they generate an approximately constant pH gradient across the cell membrane which results in an internal pH value of about one unit higher than the external one [12]. Consequently, the internal pH follows the external one without significant delay [12].

While many research groups work with *C. acetobutylicum *in batch culture, we focus on continuous culture experiments in order to investigate the effect of pH on the AB metabolism [16]. The 'forward' dynamic-shift experiment runs in the following way: after approximately 65 hours (requiring five volume changes, each after 13.3 hours) the culture is in steady state in the acid producing phase. This status can be kept for up to ten days. After this period the mega plasmid *pSol1 *which is essential for solvent production will be lost and, with it, the ability to produce solvents [18]. To initiate the metabolic shift, control of the pH of the culture via KOH is turned off. After approximately 65 hours the bacterial population is in solventogenic steady state and can, in principle, be held permanently in this state.

In this study we introduce a model at the metabolic, proteomic, and genomic levels to explain the pH-induced changes in AB metabolism and to elucidate the key genes governing this shift. We perform steady-state analyses of solventogenesis to study the effect on metabolic outputs of artificially altering gene regulation with the aim of predicting targets for optimal genetically engineered production strains with respect to butanol. The model is parameterized using data from our dynamic-shift experiments. Our findings indicate that mutations to, or otherwise altered expression of, single genes in the solvent-producing pathway are insufficient to increase significantly the butanol fermentation; a more complicated approach involving alterations in the expression of multiple genes is required.

A further important aspect of the AB fermentation is the flow of energetic and electron carriers and their availability which was investigated in the 1980 s and 1990 s by several experimental groups [19,20]. The results suggest that ATP and NADH levels might be important for the regulation of AB fermentation. Nevertheless, for this first dynamic model presented in this manuscript we do not consider this aspect in order to simplify the investigations of the pH-induced metabolic switch and its optimization with respect to butanol production.

## Methods

### Experimental setup in continuous culture

In contrast to many other groups handling batch cultures of *C. acetobutylicum*, we used continuous cultures in order to study the effect of pH on solvent production, in particular on the production of butanol. A fermentation process that can be operated continuously has several advantages over a batch process in industrial and biotechnological manufacturing: only one series of precultures is needed for a long production period, the "dead season" necessary for the filling, sterilization, cooling and clearing of the equipment is largely diminished and the volume of the fermenter vessel can be reduced without a loss of production capacity [17]. Stable solvent production can be maintained for much longer in a synthetic medium under phosphate limitation in a chemostat culture than in the traditional batch process [21].

Our model is based around continuous culture dynamic shift experiments. Experiments were performed according to a standardized experimental setup [16,18] and using standard operating procedures for extracting and handling different types of samples. The strain *C. acetobutylicum *ATCC824 was grown under anaerobic conditions at 37°C and the precultures were prepared as previously described [18]. The phosphate-limited chemostat experiments were performed in a BiostatB 1.5-l fermenter system (BBI, Melsungen, Germany) with 0.5 mM KH_2_PO_4 _and 4% (wt/vol) glucose in the supplying medium [22] and a dilution rate of *D *= 0.075 *h*^-1^. The external pH in the culture medium was adjusted to and kept constant at pH 5.7 and pH 4.5 by automatic addition of 2 M KOH. The analysis of the fermentation products was accomplished as described previously [18]. Three individual experiments were performed shifting the culture from pH 5.7 to pH 4.5 (which we call the 'forward shift' experiments) and one from pH 4.5 to pH 5.7 (the 'reverse shift' experiment). Data were taken over the full length of the observation time. The biological data is given in Additional file 1: Tables S1 - S4.

### pH-dependent modeling of the AB fermentation

As mentioned before, the metabolism of AB fermentation in *C. acetobutylicum *displays two characteristic metabolic phases, acidogenesis and solventogenesis. A detailed representation of the corresponding metabolic pathways has been published by Jones and Woods [10]. Up to the present, only a few metabolic models describing this fermentative process have been published. Papoutsakis [23] developed a stoichiometric model that could be used to estimate the rates of reactions occurring within the AB fermentation pathways of several (AB-producing) clostridial bacteria in batch culture. Importantly, however, these results are not transferable to continuous cultivations like the chemostat cultures used in this study where the cells are growing exponentially throughout. Votruba *et al*. [24] formulated a model of the fermentation process for batch cultures without including the intermediate metabolites, restricting the variables to biomass, glucose and the end products and this model captures well the two phases. Metabolic flux analysis has been applied since to the pathway [25-27]. Existing kinetic models describing the dynamics of ABE fermentation do not seek to capture the effect of pH upon the metabolic network: in Shinto *et al*. [28] (which actually considers *Clostridium saccharoperbutylacetonicum*) the switch is assumed to be glucose-dependent and enzyme levels are taken to be constant, while in Li *et al*. [29] enzyme activity is incorporated, but regulation is fed in directly to the model from experimental data (rather than being allowed to vary freely within the model or be influenced by other model components). To our knowledge, therefore, the model presented here is the first to consider the effect of pH upon the metabolic network.

#### The reduced metabolic network

Here we present a model of the AB fermentation in *C. acetobutylicum *in continuous culture. Because there is a lack of published information on the kinetic parameters governing these reactions under the conditions used in our experimental work in the literature, we aggregate a number of reactions of the metabolic network published by Jones and Woods [10]. This enables us to minimize the number of parameters that need to be estimated from the experimental work by focusing the model upon those reactions which are most likely to be regulated by the pH of the environment. Thus, as shown in Figure 1, five glycolytic steps were combined into one reaction (*R*_1_), adopting the assumption that there is a constant flux from glucose to acetyl-CoA. Additionally, we reduce the number of steps in five other reactions: the conversions of acetyl-CoA into two molecules of acetate (*R*_2_), of butyryl-CoA into two of butanol (*R*_10_), of butyryl-CoA into two of butyrate (*R*_8_), and of acetyl-CoA into two of ethanol (*R*_5_), we reduce two steps into one. Finally, we represent the three steps in the conversion of acetoacetyl-CoA to butyryl-CoA by one (*R*_9_). All intermediate reactions are listed in Table 1.

**Table 1:** Reactions that form the metabolic pathways of acidogenesis and solventogenesis and the enzymes which are required to catalyze the solvent-associated reactions, i.e. 3-7 and 9.

Reaction number	Reaction	Associated enzyme(s)
1	glucose → acetyl-CoA	
2	acetyl-CoA → acetate	
3	acetate + acetoacetyl-CoA → acetoacetate + acetyl-CoA	CtfA/B
4	acetyl-CoA → acetoacetyl-CoA	ThlA
5	acetyl-CoA → ethanol	AdhE
6	butyrate + acetoacetyl-CoA → acetoacetate + butyryl-CoA	CtfA/B
7	acetoacetate → acetone	Adc
8	butyryl-CoA → butyrate	
9	butyryl-CoA → butanol	AdhE, BdhA/B
10	acetoacetyl-CoA → butyryl-CoA	

When gene regulation is not included explicitly in the representation of a reaction, we employ Michaelis-Menten expressions. In the following section we explain our approach when gene regulation is included explicitly.

#### Incorporating gene regulation

The reactions required for solventogenesis are tightly regulated at the genetic level: production of the enzymes required to catalyze these reactions can be switched on or off (or, more generally, increased or decreased) at the transcriptional level by regulatory proteins binding at the appropriate DNA sites. The levels of the specific regulatory proteins are adjusted in response to both internal and external signals (for example pH) that are transmitted through the cell. It is therefore expected that the switch from acidogenesis to solventogenesis (or, indeed, vice versa), can be explained, at least partially, via pH-regulation of the enzyme-associated genes.

The principal enzymes involved in solventogenesis are encoded by the genes *adc*, *adhE*, *bdhA/B*, *ctfA/B *and *thlA *- see Table 1. We note that there are two *adhE *genes in *C. acetobutylicum*; the one we incorporate into our model is sometimes referred to as *adhE1*. For reasons outlined below, we include the influence of only three of these genes in our dynamic model, namely *adc*, *adhE *and *ctfA/B*.

Expression of the *ctfA/B *gene results in the acetoacetyl-CoA: acetate/butyrate:CoA-transferase [8] (or simply CoA-transferase), which is the enzyme responsible for converting acetoacetyl-CoA and the previously secreted acetate or butyrate into acetoacetate and acetyl-CoA or butyryl-CoA, respectively. The *adhE *gene is part of the same operon as *ctfA/B*, namely the *sol *operon [30]. The dehydrogenase AdhE catalyzes the conversion of butyryl-CoA and acetyl-CoA into butanol and ethanol, respectively [31,32].

The *adc *gene encodes acetoacetate decarboxylase (Adc), which is the enzyme responsible for the decarboxylation of acetoacetate into acetone and CO_2 _[14,15]. There are two known *bdh *genes: *bdhA *and *bdhB*; the products of both genes are butanol dehydrogenases [33] which (in addition to AdhE) convert butyryl-CoA into butanol. Since their roles are similar to that of AdhE their effects can be absorbed into the parameter choice for AdhE. Thus, we neglected them from our dynamic model. The gene product of *thlA *is a thiolase which catalyzes the conversion of acetyl-CoA into acetoacetyl-CoA and is therefore needed for both acidogenesis and solventogenesis [34]. It is speculated that expression of the *thlA *gene is constitutive [35] and recent experiments indicate that the difference in transcription levels of *thlA *between acidogenesis and solventogenesis is much less marked than that of *ctfA/B*, *adc*, and *adhE *[36]. Therefore, ThlA was also neglected from our dynamic model as we assume ThlA levels remain relatively constant throughout the experiments as found in [16]. The *adc*, *adhE *and *ctfA/B *genes are regulated in accordance with the metabolic phase, with their transcription increasing in the solventogenic phase [36]. Thus, they are likely to be induced via a pH-related signal and we therefore incorporate each of these genes into the model. We assume that they are each transcribed at two distinct rates: basal (*r_E _*for enzyme E, see the dashed downward arrow in (1) below) when the pH is sufficiently high (indeed, low levels of these transcripts exist before the onset of solventogenesis, see for example [37,38]) and at a higher rate (, see dashed upward arrow in (1)) when the pH of the environment is low.

We assume that all enzymatic reactions are governed by(1)

where *S *is the substrate, *E *the enzyme, *C *the complex formed by *S *and *E*, and *P *the resulting product. As in the derivation of Michaelis-Menten dynamics, we assume that the concentration of *C *is in a quasi-steady state [13,39] but, in contrast, we do not take *E *to be constant because its production is regulated by pH [13]. Thus where we wish to include enzyme concentration explicitly, we use conventional kinetic theory to obtain the following equations [40]:(2a)(2b)(2c)(2d)

where *α *= *k*_1_·*k*_2_/(*k*_-1 _+ *k*_2_), though the equations involving CtfA/B require the product of three variables in equations (2a) and (2b) since the reactions it catalyzes involve two substrates. The production of the enzyme is determined by the expression rate of the corresponding gene; as the mechanisms by which solventogenesis is induced have not been fully elucidated, we include a generic pH-dependent switch *F*(*p*) which turns on increased enzyme production below some threshold pH. The switch takes the form of the smoothed step function,(3)

where p* represents the threshold pH level around which the switch occurs and *n *dictates the steepness of the smooth switch function.

Since pH was set and controlled externally in the dynamic shift experiments, we do not introduce a differential equation for this variable. For each experiment pH is measured at regular time points. We fit a function (using 'nlinfit' in Matlab [41]) of the form

to the pH data, where *c*_1, _*c*_2 _and *c*_3 _are constants, to gain a distinct function representing pH for each simulation; this function is then fed into the model via the switch function *F*(*p*).

#### Combining the pH-dependent metabolic network with gene regulation

Where enzyme concentrations are to be included explicitly the reactions take the form outlined in the above section; otherwise Michaelis-Menten kinetics are adopted. The reactions displayed in Figure 1 and Table 1 are therefore represented by:(4)

where the limiting rate of reaction i is given by  and the corresponding dissociation constant is . We include the stoichiometric constant of two in *R*_1 _since two molecules of acetyl-CoA are formed from one of glucose. Similarly, the constant 0.5 in *R*_4 _represents the formation of one acetoacetyl-CoA from two acetyl-CoA. The resulting metabolic model is given by:(5)

where we have added to each equation an out-flow term which is the product of the dilution rate with the concentration of the corresponding metabolite because we have a constant out-flow of both extracellular and intracellular (as a result of cell out-flow) products through the chemostat.

In addition we require the following equations to represent enzyme concentrations:(6)

We fit the model to experimental data as described in the following section.

### Data normalization, parameter estimation and model simulation

**Figure 2:**
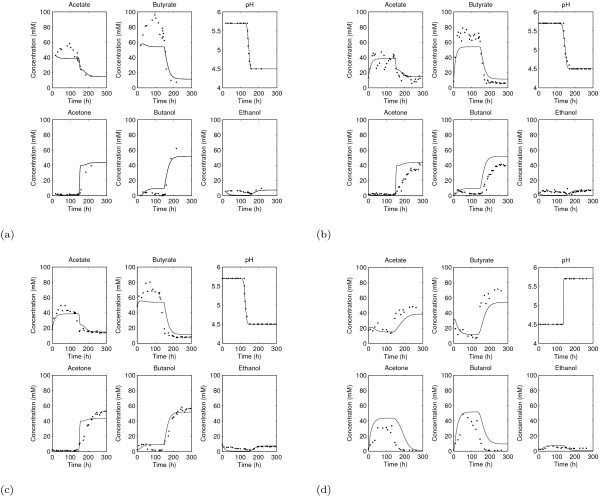
**Comparison of our model (solid lines) with the data of the dynamic shift chemostat experiments (dots)**. Figure 2(a) shows results for the 'forward' dynamic shift experiment. The two repetitions of this 'forward' dynamic shift experiment are shown in Figure 2(b) and Figure 2(c). The cells produce mainly acetate and butyrate when grown at a pH value of 5.7. During the transition phase *C. acetobutylicum *switches its metabolism (as a function of the external pH) towards the generation of the solvents acetone and butanol at a pH of 4.5. Ethanol is produced during acidogenesis and solventogenesis at approximately the same levels. In Figure 2(d) we demonstrate the comparison of the model and the data for the 'reverse' dynamic shift experiment.

All parameters are estimated from three 'forward' experiments using the SBToolbox in Matlab [42]. As mentioned before, GC data from the dynamic shift experiments are used, measuring the time until the medium reaches a certain pH level. The time span of the whole switch differed between experiments, varying from 22 ('forward' experiment 1, see Figure 2(a)) to 33.5 hours ('forward' experiment 2, see Figure 2(b)); see Supplementary File 1 for the raw data. Thus, in order to take an average of the data to be used for parameter estimation, the data had to be normalized across the dynamic shift interval to make comparisons between time points meaningful (i.e. time points for each data set should correspond to equivalent phases, namely acidogenesis, the dynamic shift, or solventogenesis). To achieve this, data sets 1 and 3 were normalized onto data set 2, i.e. the time points occurring during the dynamic shift were scaled so that the dynamic shift phase lasts 33.5 hours in all normalized data sets; solventogenesis phase time points were translated so that the start of solventogenesis occurs 33.5 hours after the start of the dynamic shift phase for all normalized data sets. Each data set was interpolated at identical time points, enabling the average of the three scaled sets to be calculated for parameter estimation, the results of which are displayed in Table 2. In all comparisons between the data and numerical solutions, the original and unscaled data sets are used.

**Table 2:** Estimated parameter values.

Parameter	Units	Value
	h^-1^	4.94
	h^-1^	2.92
	h^-1^	45.6
	h^-1^	64.8
	h^-1^	4.75
	mM	0.00158
	mM	0.00181
	mM	1.87
	mM	7.92e-006
	mM	1.40e-005
*α_3_*	mM^-2 ^h^-1^	0.00517
*α_5_*	mM^-1 ^h^-1^	0.0140
*α_6_*	mM^-2 ^h^-1^	0.00537
*α_7_*	mM^-1 ^h^-1^	4790
*α_9_*	mM^-1 ^h^-1^	347000
*r_Ad_*	mM h^-1^	0.00547
*r_Cf_*	mM h^-1^	0.000324
*r_Ah_*	mM h^-1^	0.289
	mM h^-1^	0.104
	mM h^-1^	1.06
	mM h^-1^	2.56
*n*	pH ^-1^	485
p*	pH	4.50
*D*	h^-1^	0.075

### Steady-state analysis of the metabolic network

For the purposes of developing genetic engineering strategies for enhancing butanol yield, we wish to examine changes to the steady states of the metabolic network in response to variations in transcription rates of the solvent-associated genes. Having neglected *thlA*, *bdhA *and *bdhB *from the model for studying the time-dependent dynamics in order to minimize the number of parameters to be estimated for the wild-type model, we introduce them to the steady-state studies because it is possible that overexpression or underexpression of these genes will have an effect upon the fermentation product yield. Thus, in order to explicitly vary expression of these genes, we need to derive alternative representations for the reactions in which their gene products are involved, i.e. we require parameters to represent production from each of these genes. At steady state, the concentrations of ThlA and BhdA/B are given by *r_T_*/λ and *r_B_/*λ, where *r_T _*and *r_B _*are the aforementioned production rates, the latter representing the combined levels of BdhA and BdhB (both play equivalent roles and so it is sufficient to look at them in combination). Then the rates *R*_4 _and *R*_9 _become(7a)(7b)

**Figure 3:**
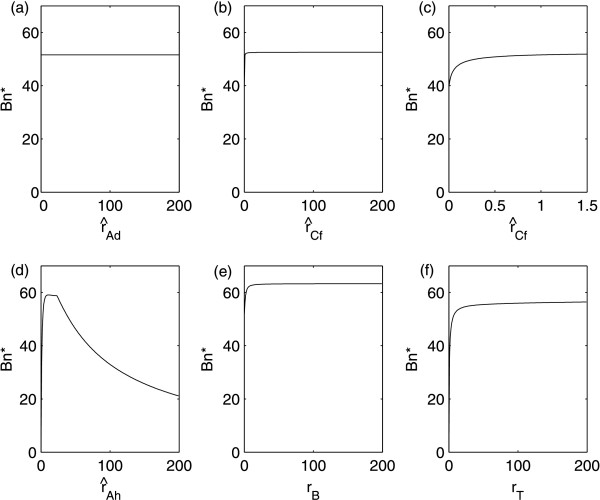
**Steady-state curves of butanol (Bn) for varying production of (a) Adc (acetoacetate decarboxylase), (b) and (c) the CtfA/B (CoA-transferase), (d) AdhE (alcohol aldehyde dehydrogenase), (e) BdhA and/or BdhB (butanol dehydrogenases) and (f) ThlA (thiolase)**. In (c) we have altered the axes of (b) in order to be able to see clearly the effect of downregulation of *ctfA/B*.

We note, however, that equation (7a) is required only when the production rate of ThlA is varied (i.e. in Figure 3(f)); in all other simulations *R*_4 _is given by the Michaelis-Menten expression in equation (4).

For simplicity, we assume that the rates of binding of butyryl-CoA with BdhA and BdhB are the same (meaning that we can consider the combined level of Bdh proteins) and that this rate is the same as that between butyryl-CoA and AdhE. We also do not include a rate of binding between acetyl-CoA and ThlA. These assumptions do not reduce the generality of the steady-state results as only the ratios of binding and production to dilution appear and no such constraints are imposed on the values of these ratios. Since we are not concerned in this section with the time-dependent behavior of the system (butanol fermentation on a large scale would presumably be carried out in a continuous culture with the system therefore at steady state), we associate a single production rate, , with each solvent-associated enzyme; this rate is different for each enzyme and is the sum of the basal production rate (*r_E _*for enzyme E), occurring regardless of pH, and the faster low-pH-induced production rate () occurring during solventogenesis, i.e. . In Table 3, we display the values of these combined enzyme production rates which correspond to those used for the wild-type-associated dynamic model.

**Table 3:** Wild-type-associated production rates (to three significant figures) for each enzyme in the steady-state investigations.

Parameter	Wild-type-associated value
	0.109
	1.06
	2.85
*r_T_*	0
*r_B_*	0

We note that *r_T _*is required only when investigating the effect of varying the production rate of ThlA.

## Results and Discussion

### Modeling and Simulation of the dynamic shift experiments

Here we apply our dynamic process-oriented model of AB fermentation by *C. acetobutylicum *that combines the metabolic network with the regulation of pH-induced proteome concentration (see Methods: pH-dependent Modeling of AB Fermentation), the pH value being the key factor for the metabolic changes in *C. acetobutylicum*. We fitted the model to experimental data of the dynamic shift experiments through the use of a switch function *F*(*p*) (see Equation (3)) to demonstrate the influence of the pH upon the fermentation products: acetate and butyrate are produced at a high pH value and acetone, butanol, and ethanol at a low pH value. The resulting numerical simulations provide a good fit to the data, see Figure 2. Thus, our model is able to explain the metabolic shift of *C. acetobutylicum *in continuous culture.

For the benefit of the reader, the SBML implementations of the model for each of the four experiments have been included as additional files. The first 'forward shift' experiment is encoded in Additional file 2, the second 'forward shift' experiment in Additional file 3, and the third 'forward shift' experiment in Additional File 4. Finally, the 'reverse shift' experiment is provided in Additional file 5.

The model is able to describe four independent experiments: three 'forward shift' experiments (Figure 2(a) - Figure 2(c)) and one 'reverse shift' experiment (Figure 2(d)). In the 'forward' experiments the culture was shifted from pH 5.7 to 4.5. In the first of these ('Forward' 1, Figure 2(a)), acidogenesis at pH 5.7 was maintained for 137 hours, after which the pH control was stopped, allowing the natural metabolic shift to the production of the solvents to begin. In this case, the shift took 22 hours, meaning that 159 hours after the start of the experiment, the cultures reached a pH of 4.5, where solventogenesis occured. The final measurement was taken at 215 hours. At this point, however, the continuous culture had not yet reached the steady state of this phase (in general C. acetobutylicum needs five complete volume changes to be in a stable state which would be after approximately 224 hours). Consequently, two repetitions of this experiment were performed, ensuring that both of these attained the steady state in each phase. In the first repetition of the 'forward' experiment ('Forward' 2, Figure 2(b)), the pH control was switched off after 137.5 hours, the system needed around 33.5 hours for the shift before reaching the solvent producing phase after 171 hours. Figure 2(b) illustrates that steady state was reached after roughly 236 hours. In the second repetition ('Forward' 3, Figure 2(c)), following 121 hours in acidogenesis, the shift lasted approximately 29 hours. From Figure 2(d), we see that the solventogenic steady state was reached after around 215 hours. In the 'reverse' experiment (Figure 2(d)) the culture was shifted from pH 4.5 to 5.7. In this case the pH control was switched off after the culture was kept for 129 hours in the solvent producing phase. The metabolic shift took around 17 hours before reaching the acid producing phase.

In each case the two distinct pH-dependent phases are evident: at pH 5.7 the main fermentation products are always the acids acetate and butyrate, while at pH 4.5 these are acetone and butanol. Due to its position in the pathway, ethanol is produced at low levels in both phases.

For the variables which are measured experimentally, initial conditions for each simulation are the corresponding first data points, and zero for all other variables. Importantly, only the 'forward' data were used to estimate parameters: no further fitting was undertaken to replicate the 'reverse' experiment in Figure 2(d). In all instances, the model describes well the experimental behavior. There are two principal discrepancies between the simulations and the data. Firstly, the model does not capture the rise in butyrate concentration which occurs during acidogenesis. It is possible that this is caused by our choice of initial conditions for the variables which are not measured experimentally (i.e. all the intermediate metabolites) since these will undoubtedly affect the dynamics of the system before reaching steady state (we will see in the subsequent section that the system is monostable meaning that the steady states in either phase will be achieved regardless of initial conditions). Secondly, rather than maintaining a relatively constant level of ethanol throughout the time course, the simulation demonstrates a switch in the amount of this metabolite being produced, the model attaining higher ethanol levels during the solventogenic phase (though the relative errors between the simulations and the data for ethanol concentration are comparable to those of the other end-products and, indeed, smaller than those of the other solvents). This second discrepancy may be more complex since the disagreement between the simulation and the data concerns the steady-state level in acid phase, rather than the dynamics. We focused on AdhE1, the major enzyme for butanol production, and did not consider AdhE2 in the model. However, recent experiments have shown that the transcript of the *adhE2 *gene is significantly upregulated during acidogenesis and antagonistically regulated with *adhE1 *[36]. Thus, it might be that this enzyme is responsible for ethanol production at higher pH values.

Our model suggests that a simultaneous change in regulation of the genes *adc*, *ctfA/B*, *adhE *is sufficient to induce the change in phenotype involving the switch between acidogenesis and solventogenesis in either direction since these are the only genes included explicitly in the model given by (5) which matches well the experimental data in Figure 2. Thus the model has helped in the characterization of the metabolic shift by identifying key genes involved in the process.

### A systems biology study of genetic engineering of the metabolic network: steady-state analysis of butanol yield

In order to investigate means of enhancing butanol yield, we use the previous model to study the effects of altered expression levels of the genes associated with solventogenesis, i.e. *adc*, *ctfA/B*, *adhE*, *bdhA/B *and *thlA*, on the steady-state butanol concentration (). A number of recent experimental studies investigate the effects of altering gene regulation on product yields [43-46], but all of these consider *C. acetobutylicum *in batch culture, rather than continuous culture, meaning that parameters and product concentrations are not directly transferable between the two experimental setups. For instance, reaction rates can be dependent upon, amongst other things, pH, and, unlike in our experiments, the pH of a batch culture is continuously evolving (in our work pH is constant in each phase). Thus, though we will make comparisons between the qualitative results of the experimental studies and our theoretical work, it must be remembered that there are fundamental differences between the two.

In order to investigate the consequence of altering gene expression, we examine the dependence of the steady states of the system on the enzyme production rates. We display the results for butanol yield in Figure 3. The results for other metabolites can be found in Additional file 1, Figures S1 - S6.

Firstly we consider , the parameter controlling production of the enzyme Adc. Adc is responsible for the conversion of acetoacetate into acetone and the analysis shows that variation in Adc levels should have a negligible effect upon butanol production, see Figure 3(a). This is consistent with results of [45] which suggested that, although required for acetone production, downregulation of *adc *has no effect upon acetone yield. These results, however, have been recently contradicted in [46] where it was demonstrated that disruption of the *adc *gene in a hyperbutanol-producing strain of *C. acetobutylicum *(EA2018) could, in fact, cause a decrease in acetone production (again in batch culture). Nevertheless, the absolute amount of butanol was not significantly changed, but consequently the ratio between butanol and acetone yield was altered. The strain variation could at least partially account for the differences in the qualitative results, in addition to a different gene manipulation technique being used (TargeTron [47], rather than antisense RNA). However, for our model to reproduce such behavior we would need to include the reverse of the CoA-transferase-mediated reaction so that the build-up of acetoacetate caused by downregulation of *adc *could be consumed in the production of end-products other than acetone. To our knowledge, the activity of the reverse reaction has not been shown to be significant (and this is why it is excluded from our model) but the results of [46] are a hint that this reverse reaction may indeed arise in certain circumstances. There are also many more factors which should be taken into account such as the evolving pH in batch culture (and the dependence of the parameters in the model upon this pH), the effect of the genetic alterations on growth rate (the disruption in the *adc *gene caused a significant inhibition on growth of the EA2018 cells) and the effect on redox balance.

The CoA-transferase (CtfA/B) synthesizes the conversion of acetate and acetoacetyl-CoA into acetoacetate, acetyl-CoA and butyryl-CoA, i.e. CtfA/B regulates the conversion of acids into solvent precursors (acetoacetate for acetone, acetyl-CoA for ethanol and butyryl-CoA for butanol). Increasing  causes an increase in the production of acetone and ethanol, while changes in butanol production are negligible, see Figure 3(b). In [45], the authors investigated downregulating *ctfA/B *(again in batch culture) with the expectation that this should lower acetone production and possibly therefore give rise to increased levels of butanol. However, it was found that downregulation of *ctfA/B *lowered levels of both acetone and butanol and this is consistent with our results, see Figure 3(c). It is speculated in [45] that the reduced butanol levels could be as a result of the antisense RNA causing unintended downregulation of *adhE*. While this is, indeed, a valid explanation, our model suggests that, at least in continuous culture, downregulation of *ctfA/B *alone could lower butanol levels because less butyryl-CoA is available from which butanol can be formed (see Additional file 1: Figure S2).

AdhE is required for two solvent-producing reactions, the conversion of acetyl-CoA into ethanol and of butyryl-CoA into butanol. We would therefore expect that increasing  (the rate of AdhE production) would increase both ethanol and butanol output. However, Figure 3(d) illustrates that this is not necessarily the case. While increasing  correlates with increased levels of ethanol, butanol levels are initially raised (coinciding with a decrease in acids) before plateauing over a range of  around the value seen experimentally in the wild-type strain (the wild-type associated value of  lies just to the left of the plateau). In this region of  we observe a sharp rise in acetate production and a smaller one in butyrate. If  is increased further, butanol levels decrease along with both acids (though the acetate levels remain high). To our knowledge, no experimental studies have been published considering the effect on product yield of *adhE *in isolation but the batch experiments reported in [44,48] examine simultaneous suppression of *ctfA/B *and overexpression of *adhE*. In this case, overexpression of *adhE *causes an increase in all solvents (though butanol levels are not increased above wild-type levels: the *adhE *overexpression compensates for *ctfA/B *downregulation). This is consistent with the lower range of  discussed above. The dual role of AdhE in solvent production obstructs the possibility of exploiting overexpression of *adhE *to increase butanol yield.

Thus we turn to BdhA and BdhB, which play a similar role to AdhE with respect to butanol production alone, and not of ethanol. Figure 3(e) demonstrates the effect of varying *r_B_*: butanol levels do increase with larger values of *r_B _*but the rate of increase decelerates with increasing *r_B_*, the levels saturating at a yield slightly higher than that seen in the model under default parameter values, i.e. those representing the wild-type cells.

The last enzyme that we consider is ThlA, which is required for the flow through the network downstream of acetyl-CoA. Figure 3(f) illustrates the consequences of varying this parameter. It is evident that, although a *thlA *mutant should be deficient in butanol, overexpressing *thlA *cannot push butanol yield significantly above that observed in the wild-type strain; this is consistent with experimental results in [43].

We are primarily concerned with genetic manipulation to maximize butanol production, this solvent being the most sought after for the manufacture of biofuels. It would be logical to predict that overexpressing the genes responsible for producing the enzymes involved in the solvent pathways of Figure 1 would increase butanol production. However, this is only true to a limited extent. The most effective way of achieving enhanced butanol yield is, according to our model, via overexpression of the *bdhA/B *genes, but the maximum levels of butanol attained via this method are not markedly higher than those observed in our wild-type experiments. Though a small increase in *adhE *transcription will induce a higher butanol yield (but, again, not much higher than that observed in the wild-type), any further increase has a detrimental effect with the increased solvent production being associated only with ethanol (shown in Additional file 1: Figure S4).

The model presented here, however, provides an efficient and simple means by which more complex approaches involving alteration to the expression of multiple genes simultaneously can be investigated. Such a theoretical approach should generate experimentally-testable hypotheses; future publications will address this.

## Conclusions

The dependence of the metabolic phase of *C. acetobutylicum *on the pH of the environment is confirmed by our experimental results: at low pH the dominant fermentation products are solvents, with acids at high pH. Incorporating gene regulation into the mathematical model of the fermentation process furnishes a mechanistic representation of this pH-induced switch between the two phases. Though the model captures well the observed behavior, including additional regulation may improve the fit. For instance, we have assumed, for simplicity, that each solvent-associated gene is activated at the same threshold pH value; assigning distinct threshold values to each gene may improve the accuracy of the model.

Systems biology approaches involving alterations to gene expression levels are frequently adopted to investigate and exploit bacteria, for example in increasing the yield of valuable fermentation products specific to the bacterium of interest, which in the case of *C. acetobutylicum *is butanol. By incorporating gene regulation into our model we are able to perform steady-state analyses to make predictions upon the likely efficacy of potential systems biology approaches. By varying gene expression level *in silico*, we infer that, to increase butanol yield, alterations in the expression of single solvent-associated genes are insufficient; a more complex approach targeting two or more genes simultaneously is required.

Kinetic models such as that presented here are a useful tool for examining gene regulation. However, the number of parameters to be measured or estimated can limit the optimal size of such a model before the level of unknown information becomes too great. Accordingly, components which could affect the behavior of the model in certain circumstances can be neglected in favor of reducing the number of parameters. Genome-scale metabolic models such as [26] and [27] can take into account the overall flux through the whole cell, but they lack key regulatory information. To gain full benefit from a systems biology approach, both types of model should be used to complement the other, e.g. the feasibility of useful mutants predicted by kinetic modeling can be tested by flux balance analysis in the genome-scale models and these genome-scale models can be used to assess the most important supplementary components to be included in the kinetic model or to identify additional sinks for existing variables. Thus, drawn together, the two modeling approaches can be mutually beneficial. The model presented in this work is therefore a stepping stone from which a theoretical exploration of gene manipulation in *C. acetobutylicum *can be conducted to generate experimentally-testable hypotheses.

## List of abbreviations

A: Acetate; Aa: Acetoacetate; AaC: Acetoacetyl-CoA; AC: Acetyl-CoA; An: Acetone; B: Butyrate; BC: Butyryl-CoA; Bn: Butanol; En: Ethanol; G: Glucose; Ad: Acetoacetate decarboxylase (Adc); Ah: Aldehyde dehydrogenase (AdhE); Cf: CoA transferase (CtfA/B); D: Dilution rate; : Limiting rate : Michaelis-Menten constant .

## Authors' contributions

Authors SH and SJ contributed equally to this work. Model formulation, parameter estimation and model simulations were performed by SH and SJ, and supervised by JRK and OW. RJF and HB lead and guided the experimental design and coordination of experiments, which were conducted by HJ. TM and JRK contributed to vital discussions. SH and SJ prepared the original draft, which was revised by JRK and TM. All authors read and approved the final manuscript.

## Supplementary Material

Additional file 1 **The file provides experimental data for each shift experiment and additional figures presenting steady-state curves for variations in the expression of genes involved in the AB fermentation pathway**. Click here for file

Additional file 2 **This SBML file encodes the model of the pH-induced metabolic shift in *C. acetobutylicum *for the first forward shift experiment**. Additionally, it is available at the JWS online model database http://jjj.biochem.sun.ac.za/. Click here for file

Additional file 3 **This SBML file encodes the model of the pH-induced metabolic shift in *C. acetobutylicum *for the second forward shift experiment**. Additionally, it is available at the JWS online model database http://jjj.biochem.sun.ac.za/. Click here for file

Additional file 4 **This SBML file encodes the model of the pH-induced metabolic shift in *C. acetobutylicum *for the third forward shift experiment**. Additionally, it is available at the JWS online model database http://jjj.biochem.sun.ac.za/. Click here for file

Additional file 5 **This SBML file encodes the model of the pH-induced metabolic shift in *C. acetobutylicum *for the reverse shift experiment**. Additionally, it is available at the JWS online model database http://jjj.biochem.sun.ac.za/. Click here for file
