# Supplementary material for: A systems biology approach to investigate the effect of pH-induced gene regulation on solvent production by Clostridium acetobutylicum in continuous culture
Source: BMC Syst Biol. 2011 Jan 19;5:10. doi: 10.1186/1752-0509-5-10 (PMC3037857; doi:10.1186/1752-0509-5-10)
Supplement: Additional file 1 — The file provides experimental data for each shift experiment and additional figures presenting steady-state curves for variations in the expression of genes involved in the AB fermentation pathway. [file 1752-0509-5-10-S1.PDF]

Experimental data

First forward shift experiment

time (hours)	ethanol (mM)	acetone (mM)	acetate (mM)	butanol (mM)	butyrate (mM)	pH
18	5.63	2.66	42.65	0	55.83	5.7
30	6.31	2.23	47.20	4.78	69.58	5.7
44	6.55	1.92	51.59	4.12	81.24	5.7
51	6.31	1.58	50.59	3.93	81.84	5.7
82	6.62	1.54	55.14	3.60	89.24	5.7
96	6.55	2.13	58.22	1.55	95.98	5.7
102	5.75	1.59	52.97	2.89	91.67	5.7
115	5.04	1.51	49.81	2.66	85.51	5.7
124	4.13	1.21	41.08	0	70.31	5.7
137	4.25	1.25	40.50	0	65.62	5.7
138	4.51	1.21	45.88	0	78.5	5.5
139.5	4.64	1.95	38.01	1.35	74.99	5.3
141	4.34	1.34	36.06	1.10	67.23	5.1
144	4.16	1.76	39.05	1.05	71.5	4.9
148	3.47	2.16	34.57	1.44	62.19	4.7
154	2.87	3.67	28.72	1.92	47.64	4.6
159	2.77	7.74	23.93	13.79	24.78	4.5
185	7.32	30.89	16.96	48.91	9.87	4.5
215	9.42	39.28	15.31	62.11	7.42	4.5

Table S1: First forward dynamic shift experiment in continuous culture.

Second forward shift experiment

time (hours)	ethanol (mM)	acetone (mM)	acetate (mM)	butanol (mM)	butyrate (mM)	pH
0	3.02	1.82	13.73	0	8.81	5.7
8	4.35	1.70	24.92	3.09	40.26	5.7
20	6.49	2.76	42.15	7.42	68.79	5.7
26	6.29	2.47	45.50	7.63	73.16	5.7
32	5.42	2.26	42.72	6.16	66.71	5.7
38	5.33	1.92	44.27	5.74	72.00	5.7
44	7.58	3.34	25.55	4.94	61.76	5.7
50	6.20	1.72	29.46	4.52	78.59	5.7
62	5.52	2.42	32.50	4.55	72.95	5.7
71	4.83	2.50	47.25	0	76.60	5.7
86	4.01	1.77	42.68	0	69.64	5.7
88	7.56	2.23	24.22	2.90	63.91	5.7
93	5.46	1.90	27.12	2.58	68.18	5.7
101	5.07	2.21	32.04	2.58	62.42	5.7
111	4.84	1.15	35.68	2.16	68.81	5.7
116	4.63	1.12	38.47	2.19	70.55	5.7
122	4.42	1.39	37.23	2.10	64.07	5.7
137.5	4.70	1.88	40.53	2.74	65.04	5.7
138.5	4.68	1.73	44.18	2.51	71.90	5.5
140	4.40	1.69	41.89	2.55	66.35	5.3
142	4.19	1.70	42.27	2.44	67.15	5.1
146.5	3.62	2.05	38.92	2.37	61.26	4.9
151.5	2.66	3.31	25.20	2.91	44.79	4.8
158.5	9.20	5.83	20.13	4.46	32.40	4.7
168	2.82	10.73	16.52	12.43	16.80	4.6
171	2.71	11.87	14.59	14.99	10.51	4.5
182	1.61	6.20	9.39	7.62	5.88	4.5
188	3.86	17.24	18.93	22.25	10.12	4.5
190	4.11	19.60	19.51	25.25	9.31	4.5
196	4.89	21.88	16.59	27.71	6.61	4.5
205	4.76	21.87	16.12	27.86	6.71	4.5
211	5.60	27.59	15.36	32.88	8.29	4.5
214	5.57	27.84	15.47	32.84	7.69	4.5
219	5.36	28.98	14.34	33.13	7.22	4.5
232	5.90	33.78	14.97	38.50	7.58	4.5
235	7.68	36.08	10.61	38.93	6.06	4.5
239	5.62	32.71	5.77	38.34	6.55	4.5
253	5.77	34.60	8.08	40.77	6.53	4.5
259	5.98	33.06	11.43	39.93	5.78	4.5
263	6.63	34.38	7.98	41.41	5.78	4.5
275	7.49	34.47	11.35	40.71	6.03	4.5
281	6.88	42.68	15.54	39.59	6.20	4.5
285	6.87	40.52	12.55	39.13	6.06	4.5
309	6.06	36.65	10.68	35.10	4.42	4.5

Table S2: Second forward dynamic shift experiment in continuous culture.

Third forward shift experiment

time (hours)	ethanol (mM)	acetone (mM)	acetate (mM)	butanol (mM)	butyrate (mM)	pH
0	9.64	2.15	18.15	2.39	48.37	5.7
9	6.05	1.87	32.89	7.90	61.80	5.7
24	5.42	1.66	39.10	9.22	59.93	5.7
33	5.57	1.43	43.99	9.32	66.00	5.7
48	5.83	1.36	49.44	9.74	78.63	5.7
57	5.27	1.24	42.65	3.19	68.08	5.7
71	4.07	1.49	49.35	7.99	80.12	5.7
81	3.80	1.29	43.00	2.26	68.59	5.7
87	3.95	1.26	42.95	2.00	70.79	5.7
99	3.58	1.29	41.70	1.84	67.39	5.7
121	3.55	1.06	41.02	1.58	64.86	5.7
122	3.50	0.99	39.12	1.84	66.38	5.5
123.5	3.25	0.87	39.39	1.66	66.35	5.3
125.5	3.42	1.56	40.12	1.65	60.40	5.1
129.5	3.42	1.56	39.62	1.61	57.95	4.9
137	2.15	2.76	29.37	2.21	40.23	4.7
150	2.23	9.32	15.40	10.20	18.84	4.5
153	2.04	11.01	16.49	13.71	12.85	4.5
172	3.09	14.92	19.28	20.48	13.53	4.5
176	3.75	19.89	16.99	26.73	10.13	4.5
194	6.41	33.65	15.80	43.12	9.95	4.5
199	6.46	35.18	15.73	44.86	9.38	4.5
202	6.42	34.62	15.19	45.29	8.77	4.5
217	6.79	41.98	14.87	50.66	7.97	4.5
220	6.46	41.86	13.97	49.06	7.41	4.5
224	6.85	45.83	14.14	52.72	7.59	4.5
241	6.94	49.25	14.74	55.46	8.15	4.5
247	7.16	51.35	16.00	58.22	8.77	4.5
250	6.66	48.82	14.48	53.97	7.86	4.5
263	6.25	47.95	13.36	51.72	7.67	4.5
268	6.54	50.65	13.88	54.76	8.00	4.5
274	6.62	51.80	13.87	55.83	8.01	4.5
287	6.62	52.70	13.60	56.36	7.94	4.5
296	6.62	52.88	14.28	56.43	8.22	4.5

Table S3: Third forward dynamic shift experiment in continuous culture.

Reverse shift experiment

time (hours)	ethanol (mM)	acetone (mM)	acetate (mM)	butanol (mM)	butyrate (mM)	pH
0	2.48	2.52	17.23	1.28	26.43	4.5
10	2.34	8.07	18.44	10.12	19.22	4.5
24	2.35	11.62	16.43	14.95	11.45	4.5
35	3.64	14.15	21.38	21.37	17.27	4.5
52	6.62	30.88	26.61	48.74	18.05	4.5
79	6.32	30.46	18.16	43.99	9.35	4.5
99	5.41	30.87	17.92	38.90	10.44	4.5
106	4.64	28.06	15.50	33.94	7.93	4.5
123	3.86	24.38	13.12	28.75	6.71	4.5
129	5.09	33.42	14.64	39.96	7.79	4.5
146	4.54	11.27	32.73	15.93	48.45	5.7
152	4.49	8.19	39.36	10.18	53.13	5.7
170	3.96	3.26	43.64	2.72	63.23	5.7
179	3.17	2.08	37.75	1.59	52.31	5.7
192	3.71	1.62	44.04	1.17	65.30	5.7
197	3.44	1.40	40.96	1.16	59.04	5.7
227	3.84	1.34	47.42	0.95	70.32	5.7
244	3.80	1.25	48.25	0.94	71.36	5.7
263	3.74	1.23	47.02	1.20	69.27	5.7

Table S4: Reverse dynamic shift experiment in continuous culture.

Additonal Figures

Steady-state curves of Acetone and Acetate for Adc

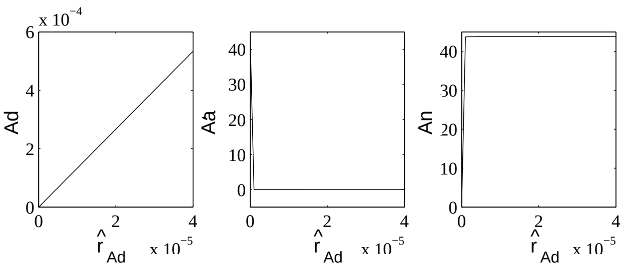

Figure S1: Steady-state curves of An and Aa for varying production of the enzyme Adc. Varying $\hat{r}_{Ad}$ has negligible effect upon the steady states of the remaining variables. The value of $\hat{r}_{Ad}$ associated with the wild-type strain (as estimated from this study) is 0.1.

Steady-state curves for CoA transferase

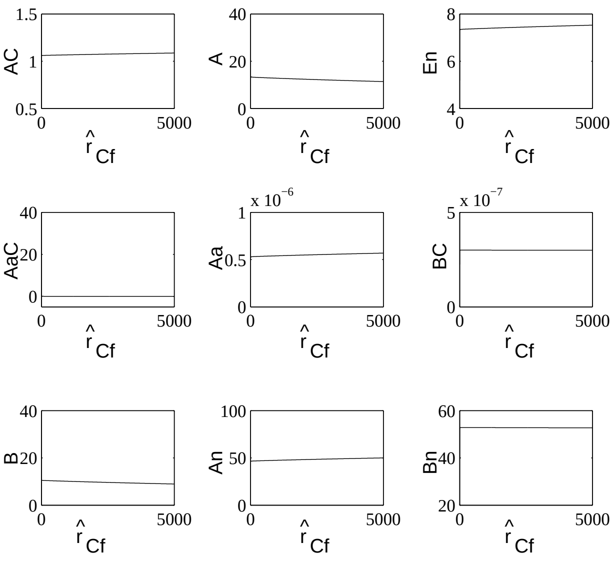

Figure S2: Steady-state curves for varying production of the CoA transferase. The value of $\hat{r}_{Cf}$ associated with the wild-type strain (as estimated from this study) is 1.1.

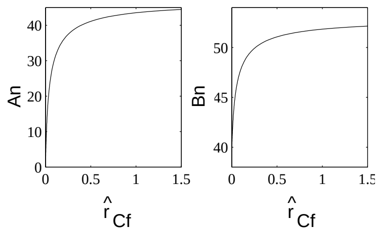

Figure S3: Steady-state curves for varying production of the CoA transferase. The value of $\hat{r}_{Cf}$ associated with the wild-type strain (as estimated from this study) is 1.1.

Steady state curves for AdhE

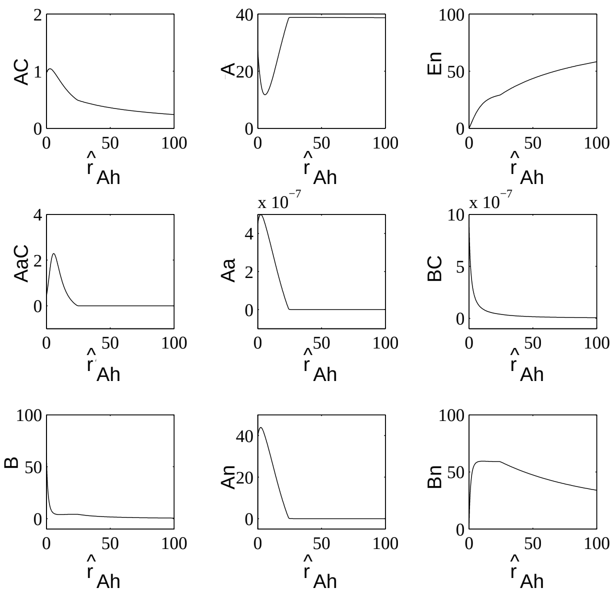

Figure S4: Steady-state curves for varying production of the enzyme AdhE. The value of $\hat{r}_{Ah}$ associated with the wild-type strain (as estimated from this study) is 2.8.

Steady-state curves for BdhA/B

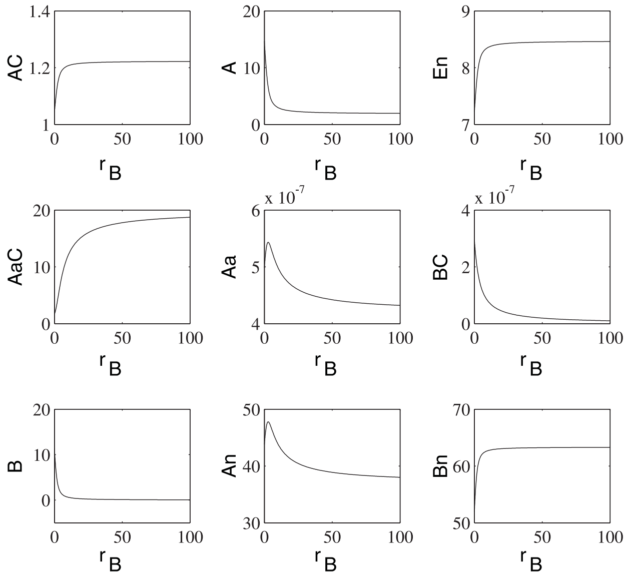

Figure S5: Steady-state curves for varying production of the enzymes BdhA and/or BdhB.

Steady-state curves for ThlA

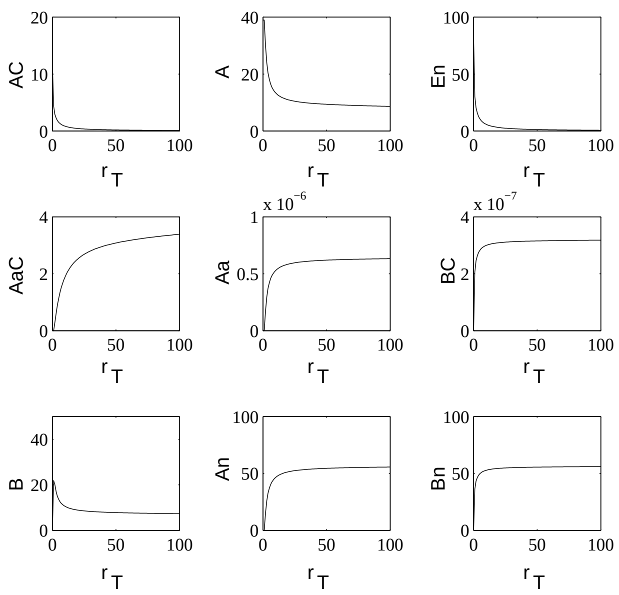

Figure S6: Steady-state curves for varying production of the enzyme ThlA.
